# Assessing Genotoxicity and Cytotoxicity Induced by X-Ray Exposure From Cone Beam Computed Tomography at Varied Fields of View

**DOI:** 10.7759/cureus.66459

**Published:** 2024-08-08

**Authors:** Chintha Vishwadha, Janumpally Varshitha Thanmai, G Ramlal, Srikanth Goud G, Tejaswi Katne, Poreddy Vaishnavi Reddy

**Affiliations:** 1 Department of Oral Medicine and Radiology, SVS Institute of Dental Sciences, Mahabubnagar, IND; 2 Department of Oral medicine and Radiology, SVS Institute of Dental Sciences, Mahabubnagar, IND

**Keywords:** field of view, oral exfoliated cells, cytotoxicity, genotoxicity, cbct

## Abstract

Introduction: The practice of dentistry benefits greatly from cone beam computed tomography (CBCT) and advantages should be prioritized over hazards; even modest doses of X-rays have the potential to have cytotoxic effects, damage DNA through their clastogenic impact, and stimulate the creation of micronuclei along with further nuclear changes.

Aims and objectives: To assess the genotoxicity and cytotoxicity of X-rays in exfoliated oral mucosal cells from patients who underwent CBCT scans at different fields of view (FOV), and to examine and assess the extent of cytotoxicity and genotoxicity caused by X-rays in oral exfoliated cells of people who were subjected to CBCT at different fields of view (FOV).

Material and methods: Following CBCT exposure, 66 patients were chosen from the Department of Oral Medicine and Radiology at the SVS Institute of Dental Sciences, Mahbubnagar. Cells from the buccal mucosa were then extracted using the exfoliative cytology method, and the samples were examined under a microscope to look for nuclear and cytological abnormalities.

Results: A paired t-test analysis revealed that mean micronuclei increased significantly in each study group from before to after exposure. It increased in Group I from 93.59 to 96.05, in Group II from 83.27 to 91.86, and in Group III from 86.05 to 97.00. Various test analyses revealed an important relation between exposure status and the presence of karyorrhexis in Group III. There was no association in other groups.

Conclusion: The study revealed a significant increase of micronuclei in subjects after exposure to radiation at various FOVs. There was an increased karyorrhexis following radiation exposure in all groups at various FOVs. The significant association between exposure and karyorrhexis in the larger size FOV group was noticed further potentiating the extent of increased damage as the size of FOV is increased.

## Introduction

Over the past three decades, various fields of dentistry have made great advancements. With these developments, there is an increased requirement for greater accuracy in diagnostics, particularly imaging techniques, from straightforward intraoral peri-apical radiographs to digital radiography as well as other sophisticated imaging methods [1]. Switching from analog to digital radiography has made these processes faster and simpler, as well as making it easier to store, manipulate (brightness/contrast, crop images, etc.), and retrieve images [2]. The transition from a 2D to a 3D paradigm has improved pathology diagnosis accuracy and increased accessibility to craniofacial structures for testing [3].

Research in the literature evaluated the degree of DNA damage and the genotoxic consequences in human tissues based on the frequency of micronuclei [4]. The mucosal epithelium of the mouth can be regarded as the preferred tissue for X-ray effect analysis as it is directly exposed to radiation, making it a prime target for radiation-induced damage during a cone beam computed tomography (CBCT) scan [5,6]. This study aims to evaluate the genotoxicity and cytotoxicity of X-rays at various fields of view (FOV) using CBCT. It specifically looks at how X-rays affect the cells of patients receiving CBCT at various FOVs [7]. Hence the study aimed to assess and examine the genotoxicity and cytotoxicity of X-rays in exfoliated oral mucosal cells from people who underwent CBCT scans at different FOVs.

## Materials and methods

Sixty-six participants in the study underwent CBCT radiodiagnosis at the Department of Oral Medicine and Radiology, SVS Institute of Dental Sciences, Mahabubnagar. Following the procedures mandated by the institutional ethical committee, the study proceeded only after the individuals gave their informed permission. Based on varying FOVs used during CBCT, the individuals were divided into three groups: Group I with 22 subjects underwent exposure to a local FOV (5x5cm), Group II where 22 subjects underwent exposure to a standard FOV (16x10cm) and Group III where 22 subjects underwent exposure to a stitching FOV (16x18cm). A sample size of 66 (per group 22) is calculated for an effect size of 0.40 an alpha error of 0.05 and a power of 0.80. The age range in the study sample was 16 to 66 years.

Participants who were of either gender and age with intact oral mucosa requiring CBCT scans in any of the craniofacial regions included in the study. Those with any oro-mucosal lesion or an autoimmune disorder affecting the oral mucosa and who were undergoing radiotherapy or chemotherapy were excluded [8-10].

Every CBCT scan was ordered by a dentist and carried out using a Dentium machine (Dentium Picasso Trio, Dentium Co., Ltd., Seoul, Republic of Korea) with a standard 90 kV and 8 mA in a 16 x 18 cm FOV, with a unit of radiation exposure as a gray unit according to all accepted projection criteria and protocols. Three distinct protocols were utilized with this scanner: local, standard, and stitching, according to the dentist's desire, on the dental-maxillofacial area [11].

Preparing slides and collecting cells

**Figure 1:**
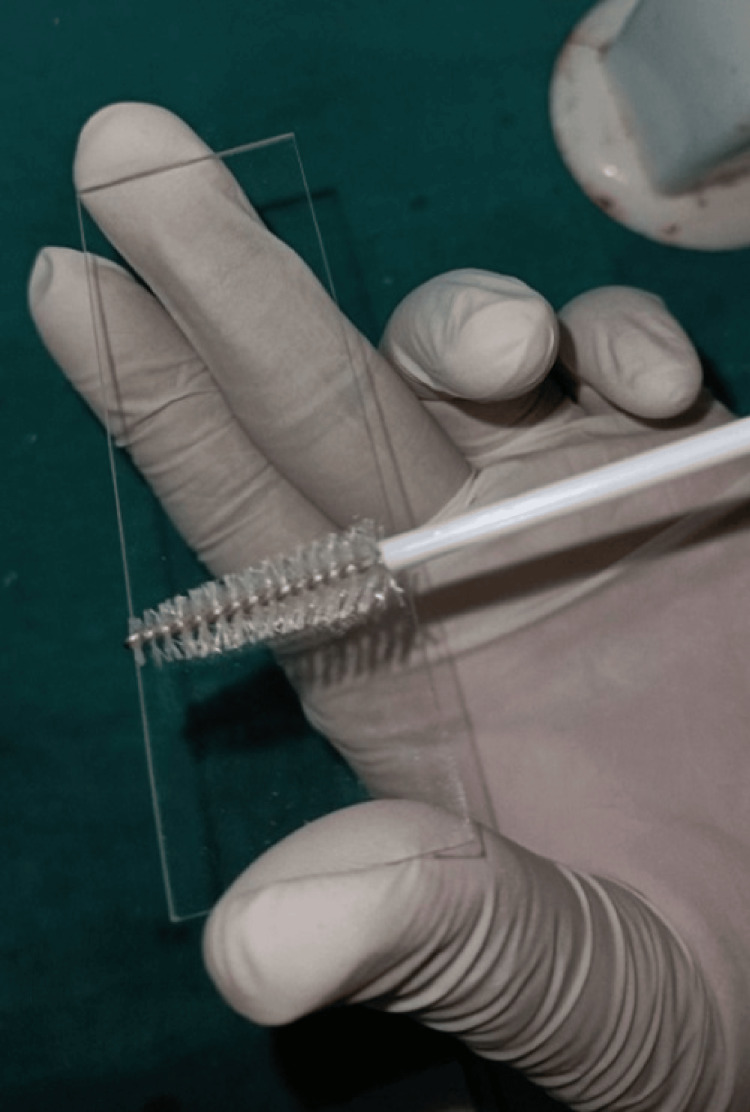
Collection of exfoliated buccal mucosal cells using a cytobrush which transfers exfoliated buccal mucosal cells onto slide

Exfoliated mucosal cells were collected immediately before CBCT treatment and again after 10 days following the exposure [12]. The timeframe of 10-14 days for conducting examinations and studies on the buccal mucosa post-radiation exposure is crucial due to the regenerative nature of the tissue. The epithelial cells in the buccal mucosa undergo turnover approximately every 10-14 days, during which new cells replace the old ones. This turnover period allows the damaged layers of cells, which have been exposed to radiation, to be scraped easily [12]. After using mouthwash, the participants were instructed to rinse their mouths with water to ensure a clean sample collection environment. A cytobrush was then utilized by trained staff to gently scrape and collect exfoliated cells from the bilateral buccal mucosa. These cells were then transferred onto a slide for further analysis. Slides were stained using the Papanicolaou method (Pap stain) after being moved to a jar with a fixing solution [13]. To ensure consistency and minimize procedural errors, we have chosen to stain the controls using the same Pap stain. This approach helps maintain uniformity in staining protocols and facilitates accurate comparative analysis between samples and controls (Figure 1).

Cytological analysis

An expert oral pathologist randomly inspected the slides. Using a 400x magnification transmitted light microscope, the frequency of micronucleated buccal mucosa cells and karyorrhexis was ascertained. The micronucleated cells are scored for the study of DNA damage (genotoxicity), and the karyorrhexis was taken into consideration for the evaluation of cell damage (cytotoxicity). The criteria for abnormality of micronucleus formation that were followed in the study were as follows: (1) less than one-third of the diameter of the primary nucleus; (2) colour similar to or lighter than the nucleus, which helps in excluding large staining particles; (3) located further away from the smaller nucleus by three or four nucleus diameters (4); not touching the nucleus and not more than two micronuclei associated with a nucleus. 

**Figure 2:**
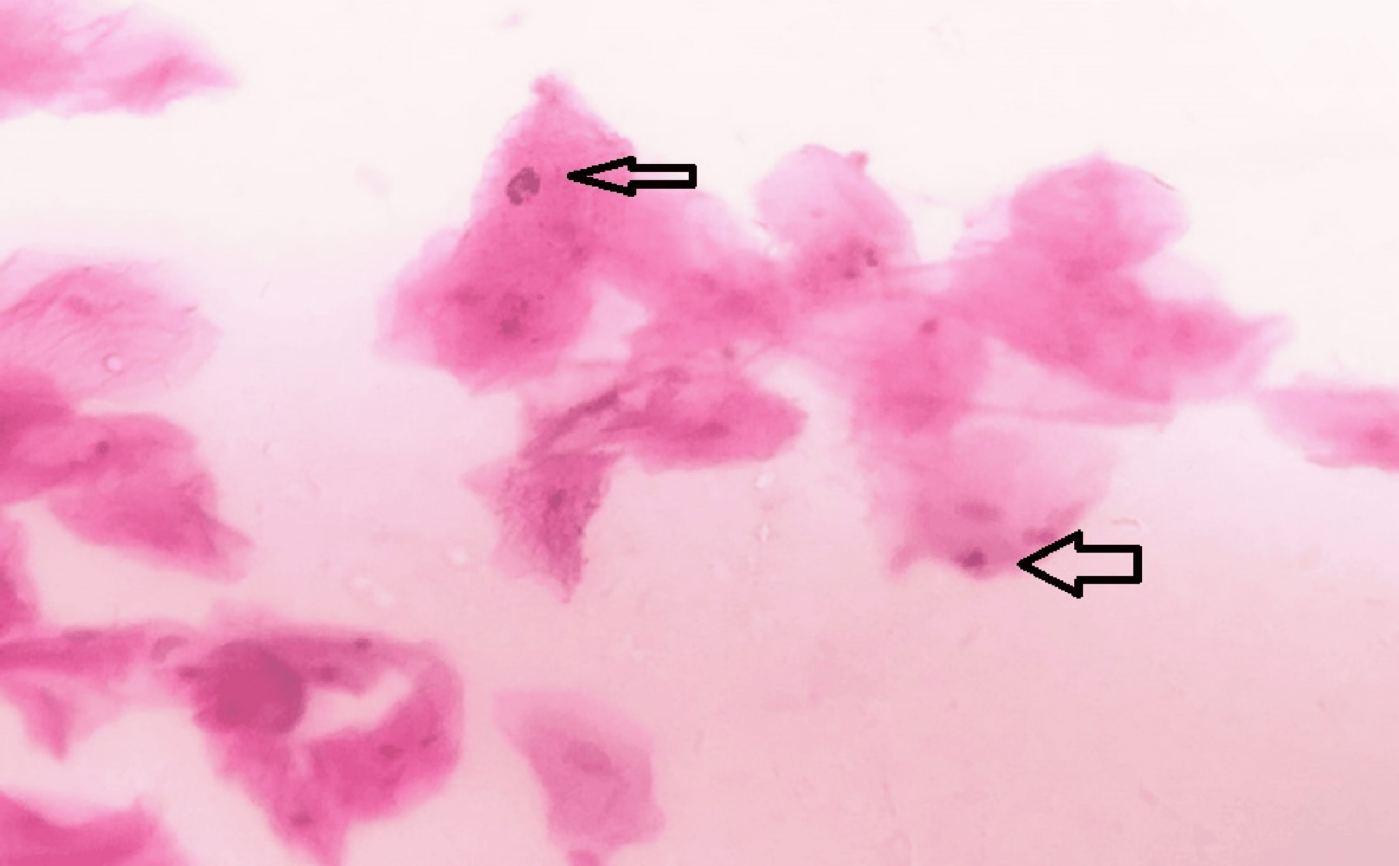
Histopathological picture showing micronuclei

The following nuclear anomalies in sample smears were noticed: (1) binucleates, which involve the presence of two nuclei within a cell, and (2) karyorrhexis, referring to nuclear disintegration involving loss of integrity of the nucleus. A total of 1000 cells were analyzed from each patient for each sampling time (before and after CBCT exposure) (Figure 2).

Data analysis

Results were obtained and then reported as percentages (%). Using analysis of variance (ANOVA), intergroup analysis was carried out. P-values less than 0.05 are regarded as statistically significant [14]. 

## Results

In the present study, the analyzed data in Table 1 revealed that there were 39 males (59.1%) and 27 females (40.9%).

**Table 1:** Stratification of the sample by gender

Gender	Frequency	Percent
Male	39	59.1
Female	27	40.9
Total	66	100.0

One-way ANOVA analysis of data before exposure to radiation revealed that the mean values of micronuclei before exposure to radiation in Group I were 93.59, in Group II, 83.27, and in Group III, 86.05 (Table 2).

**Table 2:** Genotoxicity among different groups - before exposure to radiation P-values less than 0.05 are regarded as statistically significant SD: standard deviation

Variables	N	Mean micronuclei	SD	F-value, degree of freedom	P-value
Group I	22	93.59	17.614	3.070, 2	0.053
Group II	22	83.27	13.083		
Group III	22	86.05	11.470		
Total	66	87.64	14.743		

One-way ANOVA analysis of data after exposure to radiation revealed that the mean value of micronuclei after exposure to radiation in Group I was 96.05, in Group II was 91.86, and in Group III was 97.00. It was also revealed that mean micronuclei didn’t differ significantly after exposure to radiation between study groups, with mean micronuclei of 94.97 and a p-value of 0.502 (Table 3).

**Table 3:** Genotoxicity among different groups - after exposure to radiation P-values less than 0.05 are regarded as statistically significant SD: standard deviation

Variables	N	Mean micronuclei	SD	F-value, degree of freedom	P-value
Group I	22	96.05	17.581	328.394, 2	0.502
Group II	22	91.86	15.015		
Group III	22	97.00	13.104		
Total	66	94.97	15.272		

Paired T-test analysis of data before and after radiation exposure revealed that mean micronuclei increased significantly in each study group from before to after exposure. It was increased in Group I from 93.59 to 96.05, in Group II from 83.27 to 91.86, and in Group III from 86.05 to 97.00 (Table 4).

**Table 4:** Genotoxicity among different groups - before and after radiation exposure P-values less than 0.05 are regarded as statistically significant SD: standard deviation

Group	Variables	Mean	N	SD	Std. Error Mean	P-value
Group I	Micronuclei before exposure	93.59	22	17.614	3.755	0.0001
	Micronuclei after exposure	96.05	22	17.581	3.748	
Group II	Micronuclei before exposure	83.27	22	13.083	2.789	0.0001
	Micronuclei after exposure	91.86	22	15.015	3.201	
Group III	Micronuclei before exposure	86.05	22	11.470	2.445	0.0001
	Micronuclei after exposure	97.00	22	13.104	2.794	

Regarding cytotoxicity among different groups, in Group I karyorrhexis was evident both before and after radiation in 7 of the 22 subjects, in Group II it was evident both before and after radiation in 7 of the 22 subjects, and it was evident both before and after radiation in 6 of the 22 subjects in Group III (Table 5).

**Table 5:** Cytotoxicity among different groups – before and after exposure to radiation FOV: Field of view P-values less than 0.05 are regarded as statistically significant

Size of FOV	Karyorrhexis	
	Before exposure to radiation (Gray units)	After exposure to radiation (Gray units)
Group I	7	13
Group II	7	18
Group III	6	21

The chi-square test revealed a significant relationship between exposure status and the presence of karyorrhexis in Group III. This association was not seen in other groups (Table 6). 

**Table 6:** Cytotoxicity between different groups – before and after exposure to radiation P-values less than 0.05 are regarded as statistically significant

GROUP			Karyorrhexis after radiation (Gray unit)		Total	Χ^2^	P-value
			Present	Not present			
Group I	Karyorrhexis before radiation (Gray unit)	Present	7	0	7	0.393	0.531
		Not present	6	9	15		
	Total		13	9	22		
Group II	Karyorrhexis before radiation	Present	7	0	7	0.841	0.359
		Not present	11	4	15		
	Total		18	4	22		
Group III	Karyorrhexis before radiation	Present	6	0	6	7.108	0.008
		Not present	15	1	16		
	Total		21	1	22		

## Discussion

Determining the genotoxic effects of X-rays is important since there is considerable evidence linking genetic damage to the development of cancer [15]. It is well acknowledged, therefore, that radiation dosages are never safe and that exposures will eventually accrue biological damage. For many years, cytogenetic biomonitoring has been utilized in the health sciences to assess risk, diagnose and stage illnesses, and offer information on susceptibility status and risk levels [16]. It is employed in estimating the danger that human populations will face from exposure to harmful substances. This test has been used to detect cell radiosensitivity, evaluate genetic damage following occupational exposure to X-rays, and investigate the persistence of chromosomal abnormalities [17-20].

This finding supported the theory that X-rays are cytotoxic and that their effects are mostly dependent on the dose and frequency of usage in addition to the individual's capacity to heal such damage [21]. The fact that direct X-ray irradiation of the buccal mucosa causes direct biologic damage can be used to explain the increased micronucleus frequency in exfoliated buccal mucosal cells found in this investigation following exposure [22,23].

Thus, it seemed from the study that there was no meaningful correlation between radiation exposure and genotoxicity. Since some micronucleated cells eventually die following a cytotoxic shock, it may be stated that cytotoxicity interferes with the formation of micronuclei. This further supports the idea that X-rays do not have a mutagenic impact, although bigger sample sizes might be used in future studies in this area [24-27]. Despite the fact that previous research has revealed the cytotoxic and genotoxic effects of X-rays, this study is unique in that it critically assessed the effects of CBCT at different FOVs. The micronucleus test, which can detect the effects of low radiation doses, appears to be adequately verified and highly sensitive when applied to exfoliated human buccal mucosa cells. The investigation assessed the differences in nuclear morphology, which is thought to be a pathogenic indicator of cancerous cells [28,29].

Given that the results of this study were significant and achieved statistical significance, bigger sample sizes and longer follow-ups are needed for future research. Since the current study solely assessed the negative effects of CBCT, the cytotoxic and genotoxic effects of X-rays from different dental radiology imaging methods may be objectively evaluated. Further study might compare the cytotoxic and genotoxic effects of X-rays with different imaging modalities. The findings may be further validated by taking into account different methods (especially those that can identify DNA adducts, single and double-strand breaks, point mutations, and others) for evaluating the genotoxicity brought on by dental X-rays [30-35]. The 66 participants in the current research ranged in age from 16 to 66, with a mean age of 39.33 years. The mean age of the sample was reported to be 26±9.18 years in research by Ribeiro et al. [32], which is approximated to be 26 years in the current investigation. However, the study conducted by Cerqueira et al. [33] on patients subjected to panoramic dental radiographs with a mean age of 24±1.023 years was in contrast to the present study. In the present study, data analysis revealed that there were 39 males (59.1%) and 27 females (40.9%), which indicates a higher number of males in the sample. This was in contrast with the study conducted by Anuradha et al. [34] on 50 healthy individuals subjected to digital panoramic radiography for diagnostic purposes, which reported that females were more likely than males. Cytotoxicity was significantly increased in the only large FOV group and it may be due to differences in radiation dose and site of collected cells [24]. These results were in contrast with the study conducted by El-Ashiry et al. [23] on subjects who underwent panoramic dental radiography. Results showed that dental panoramic radiography has the potential to cause genotoxicity and chromosomal harm in youngsters.

The study's limitations include a relatively small sample size of 66 patients. This sample size may restrict the generalizability of findings to broader populations or other clinical settings. Conducted at a single institution, the research may reflect specific patient demographics, radiation protocols, and local biases, potentially limiting its applicability beyond this setting. Selection bias might affect the results, as participants were selected from those already undergoing CBCT scans, possibly restricting the representation of the general population and impacting external validity. 

## Conclusions

In conclusion, this study underscores the pressing need to evaluate the genotoxic and cytotoxic effects of X-ray exposure in individuals undergoing cone-beam computed tomography (CBCT) across various fields of view (FOVs). The findings reveal a significant increase in micronuclei frequency and karyorrhexis in oral exfoliated cells following radiation exposure, indicating a potential risk of DNA damage and cellular toxicity associated with CBCT imaging. A notable correlation between exposure and karyorrhexis was observed in subjects undergoing CBCT with larger FOVs, suggesting an amplified risk of radiation-induced damage with increased FOV size.

Despite the limitations of this study, such as sample size and follow-up duration, the results provide valuable insights into the potential hazards of CBCT imaging and emphasize the importance of optimizing radiation protocols and implementing preventive measures to minimize the adverse effects on oral mucosal cells. Additionally, future research should focus on larger sample sizes, longer follow-up periods, and comparative analyses with other dental radiological imaging modalities to comprehensively assess the cytotoxic and genotoxic effects of X-ray exposure in dental practice.
